# Effectiveness of family psychoeducation for major depressive disorder: systematic review and meta-analysis

**DOI:** 10.1192/bjo.2022.543

**Published:** 2022-08-02

**Authors:** Fujika Katsuki, Norio Watanabe, Atsurou Yamada, Takaaki Hasegawa

**Affiliations:** Department of Psychiatric and Mental Health Nursing, Nagoya City University Graduate School of Nursing, Nagoya, Japan; Department of Psychiatry, Soseikai General Hospital, Kyoto, Japan; Department of Psychiatry and Cognitive-Behavioral Medicine, Nagoya City University Graduate School of Medical Sciences, Nagoya, Japan; Center for Psycho-oncology and Palliative Care, Nagoya City University Hospital, Nagoya, Japan

**Keywords:** Major depressive disorder, family psychoeducation, randomised control trial, meta-analysis, systematic review

## Abstract

**Background:**

Although its effect has not been verified, family therapy – such as family psychoeducation (FPE) – is a widely used intervention for treating major depressive disorder (MDD). To our knowledge, no systematic review and meta-analysis exists that examines the effect of FPE on MDD.

**Aims:**

To assess evidence on the effectiveness of FPE on depressive symptoms in people with MDD.

**Method:**

We searched several databases – including PubMed, MEDLINE and Web of Science, among others – to identify eligible studies on the topic published up to March 2022. Our criteria included studies on participants with a primary MDD diagnosis and their family members and excluded studies on people with bipolar disorders and other mental illnesses. In the included studies, family members in the control groups did not receive FPE. Participants in both the intervention and control groups received standard treatment. Two researchers independently selected relevant publications, extracted data and evaluated methodological quality using the Cochrane risk of bias assessment tool and GRADE evaluation. The protocol was registered with PROSPERO (no. CRD42020185884).

**Results:**

The meta-analysis included five studies with 301 patients with MDD and their family members. The effect of FPE on patients’ symptom severity, compared with the control condition, at 16 weeks was available for five comparisons of four randomised control trials (RCTs); a final follow-up was available for six comparisons of five RCTs. The meta-analysis showed a statistically significant improvement in patients’ symptoms, compared with control, at 16 weeks (s.m.d. = −0.52, 95% CI −1.03 to −0.01) and at a final follow-up (s.m.d. = −0.53, 95% CI −0.98 to −0.08). The meta-analysis on the effect of FPE on family functioning showed a non-significant improvement both at 16 weeks and at final follow-up.

**Conclusions:**

FPE had a small but statistically significant effect on depressive symptoms in people with MDD, in both the short and long term. However, according to the GRADE framework, all outcomes are graded very low on certainty; therefore, more high-quality research is needed.

Major depressive disorder (MDD) is a common mental illness: combined study data from 30 countries show 1-year and lifetime prevalence rates of 7.2% and 10.8% respectively.^1^ Moreover, it has a continuous high risk of recurrence, which represents an increased disease burden.^2^ Findings from the STAR*D (Sequenced Treatment Alternatives to Relieve Depression) study suggest that 30% of people with MDD do not enter remission, despite receiving fourth-line treatment.^3^ Additionally, 50% of people with MDD relapse after their first episode, 70% after the second and 90% after their third.^4^ This illustrates the real danger of chronic depression after the initial MDD diagnosis.

## Effects of MDD on families

For many families, an MDD diagnosis means long and difficult periods marked by high stress. It can involve a great deal of familial suffering, including a higher divorce rate^5^ and severe financial strain.^6^ Balkaran et al's study^7^ of five Western European countries reported that caregivers of adults with unipolar depression have a greater burden than caregivers of adults with other chronic mental or physical diseases (e.g. Alzheimer's disease, bipolar disorder, schizophrenia, cancer or chronic kidney disease). In terms of their health status (measured using the Short Form-6 Dimensions) and health-related quality of life (measured using the Medical Outcomes Study 12-item Short-Form survey instrument, version 2), the unipolar depression caregiver group had significantly worse scores than the other chronic disease caregiver groups. Families of patients with MDD find some patient behaviours difficult to understand, and they experience negative consequences such as grief, withdrawal and worrying, which cause further problems; however, few families know how to manage patients’ difficult behaviour.^8^ Marguerite et al^9^ reported that caregivers’ coping strategies, such as problem-solving, positive thinking and avoidance, affect both their own and patients’ mental health. Caregivers’ use of positive thinking and problem-solving was associated with a decrease in their own level of anxiety. Conversely, using avoidance strategies increased caregivers’ own depression and anxiety, as well as patients’ anxiety. These results suggest that it is important for both patients and caregivers to have the appropriate information on how to treat depression and to implement appropriate coping strategies for daily problems in order to recover from depression and to maintain the caregiver's own mental health.

## Expressed emotion

A family's expressed emotion (EE) is a good predictor of schizophrenia relapse.^10,11^ EE is an index representing the familial relationship and is assessed by examining the content of emotions expressed towards the patient by family members. Concerning MDD, although Hayhurst et al^12^ reported that there is no clear association between the EE of a spouse and recurrence of depression in the patient, three studies reported that high EE predicts negative consequences.^13–15^ A study involving 39 people with MDD reported that 59% of those living with high-EE spouses relapsed, whereas none living with low-EE spouses did so.^13^ Among 40 individuals with MDD, another study's logistic model indicated that two strong factors predicted the 6-month outcome of depressive episodes: the level of criticism in the family's EE and their history of major depression.^15^ In a study on the interaction between individuals with MDD and the level of their spouses' EE, compared with low-EE spouses, spouses rated as high EE expressed more negative (and fewer positive) feelings towards partners with MDD, both verbally and non-verbally. High-EE spouses also made more critical comments and disagreed with partners more frequently. Moreover, high levels of EE in spouses were associated with low frequencies of self-disclosure in patients.^16^ These studies suggest the need for a more family-oriented approach in MDD treatment. UK clinical guidelines from the National Institute for Health and Care Excellence (NICE) recommend a couples-focused intervention as one of the evidence-based treatments for depression.^17^ However, Henken et al's review reported that despite the lack of high-quality evidence in this field, family therapy is already a widely used intervention for treating depression.^18^

## Family psychoeducation

Family psychoeducation (FPE) is recognised as an important part of optimal treatment, along with traditional medication and counselling, for people with a psychotic disorder.^19,20^ This intervention is recommended for schizophrenia treatment by the US Department of Health and Human Services’ Substance Abuse and Mental Health Services Administration (SAMHSA)^21^ and by NICE.^22^ As treatment for bipolar disorder, FPE – called family-focused therapy – has been shown to be effective as an adjunctive treatment.^23,24^ FPE is the method of working with families who want to support persons with mental illness. FPE is more than merely information provision; it ensures that people understand the illness. Importantly, it focuses on the development of problem-solving, communication and coping skills and the enhancement of social support to manage mental illness. In family therapy, the family itself is the object of treatment, whereas in the FPE approach, the illness is the object of treatment, not the family.^21^ This intervention has been shown to reduce the relapse rate and hospital admissions among individuals with psychotic disorders and to reduce caregiver burden.^23–26^

However, FPE is still not widely available for people with MDD and their families. Several studies report on the effectiveness of FPE for MDD. Shimazu et al^27^ examined the effect of FPE in treating 25 individuals diagnosed with MDD, compared with a control group (*n* = 32). The FPE consisted of four sessions for caregivers without the participation of patients. The findings showed that FPE was effective in preventing relapse at 9 months in adults with MDD. Fiorillo et al^28^ and Luciano et al^29^ reported similar findings from their study. Their intervention package consisted of 12 single-family sessions. The authors reported that the FPE intervention helped reduce personal and family difficulties caused by depression and improved social contact. However, the follow-up period in this study was short (6 months), with no clarity on long-term effects. Clarkin et al^30^ and Glick et al^31,32^ compared a psychoeducational in-patient family intervention with standard hospital treatment of depression symptoms. Female participants in the experimental group reported a reduction in depression symptoms. This study revealed gender to be a mediating variable.

There is, to date, no meta-analysis examining the effectiveness of FPE for MDD, and only one related narrative review could be traced.^33^ Our clinical question therefore asks whether FPE reduces the severity of symptoms in people with MDD, compared with a control group. This systematic review and meta-analysis aims to evaluate the evidence on the effectiveness of FPE on the severity of depressive symptoms in people with MDD.

## Method

A review protocol was developed following the Preferred Reporting Items for Systematic Reviews and Meta-Analysis (PRISMA) guidelines^34^ and was registered with PROSPERO (reference: CRD42020185884).^35^ We searched the PubMed, MEDLINE, Embase, PsycInfo, the Cochrane Library, Emcare, ProQuest Dissertation and Theses, and the Web of Science databases to identify eligible studies of FPE for MDD, from their inception up to March 2022. The search included all relevant terms, such as ‘family education’, ‘family psychoeducation’, ‘family’, ‘family therapy’, ‘family relations’, ‘family health’, ‘health education’, ‘patient education as topic’, ‘psychoeducat*’, ‘psycho educat*’, ‘family therap*’ and ‘family intervention’, in combination with ‘depressive disorder’, ‘depressive disorder*’ and ‘depression’ (supplementary Table 1, available at https://doi.org/10.1192/bjo.2022.543). Abstracts identified during the literature search were screened by two pairs of review authors (F.K. and A.Y., F.K. and T.H.) independently. Potentially eligible articles were read by the two pairs of review authors (F.K. and A.Y., F.K. and T.H.) to determine whether they met the eligibility criteria. Disagreements were discussed with a third review author (N.W.) until consensus was reached. As necessary, additional information was obtained from the study authors.

### Eligibility criteria

#### Study types

All published and unpublished randomised controlled trials (RCTs) were included. We excluded quasi-randomised studies, such as those that allocated participants according to alternate days of the week. When identified, we included cluster trials. We also included cross-over trials that were identified, but only used data up to the first cross-over because of the instability of outcomes and the likelihood of carry-over effects of all treatments.

#### Participants

The study populations included patients with a primary diagnosis of MDD, based on DSM or ICD criteria, and their family members. There were no restrictions on participants’ gender, ethnicity or comorbidities. Patients with MDD who had no current symptoms and those in both hospital and community settings were included. The following studies were excluded: those with patients whose main diagnosis was schizophrenia, bipolar disorders, Alzheimer's disease, anorexia nervosa, bulimia nervosa, psychotic episodes or epilepsy; those with patients with MDD secondary to physical illness; studies in which patients who were less than 18 years old accounted for more than 50% of the sample; and studies in which the only family members attending the FPE were children younger than 18 years.

#### Intervention

FPE is a method for working with families who are supporting persons with mental illness. FPE comprises several established methods, such as family management,^36^ behavioural family therapy^37^ and multiple-family therapy.^38^ These interventions often overlap with each other. The common elements in these interventions are as follows: providing education on the nature of mental illness; giving practical advice on coping strategies; encouraging families to sustain their own life trajectories; teaching effective communication skills; enhancing communication and assisting family members in using modes of problem-solving and stress management.^39^ Considering these elements, we defined FPE as follows: FPE is more than just providing information; it ensures that affected people understand the illness. It also focuses on the development of problem-solving, communication skills, coping skills and social support enhancement to manage depression. We included individual and group programmes that involve interaction between the information provider and participants. Any delivery model was included, such as face-to-face, online virtual forums (such as phone, chat or Skype) and a mix of different delivery models. We excluded brief interventions that focus only on didactic education or health information using textual or video materials in any delivery model. There were no restrictions on programme duration or on the number of sessions. The programmes were led by medical professionals, but there was no limit on the training time of the facilitators delivering the psychoeducational intervention. Mutual support groups that were facilitated solely by family members from the outset were excluded.

#### Comparators

Family members in the control group did not receive FPE. They might have received other support (such as counselling or self-help support groups) but were excluded if they received support with treatment components of psychoeducation. Control groups on a waiting list and with active placebo were included. Studies with structured psychotherapy for only intervention group patients were excluded.

### Outcomes

The primary outcomes of this study were patients’ depressive symptoms, depression above the threshold and family functioning. Assessment times were divided at 16 weeks (defined from 12 weeks to 20 weeks) and a final follow-up. We defined each primary outcome as follows.

- Patients’ depressive symptoms were measured on the following standard scales: masked (blind)-assessed Hamilton Rating Scale for Depression (HRSD),^40^ which was preferentially adopted; masked-assessed Montgomery–Åsberg Depression Rating Scale (MADRS);^41^ the self-reported Beck Depression Inventory (BDI);^42^ and the self-reported Patient Health Questionnaire (PHQ-9).^43^
- For ‘depression above the threshold’, a cut-off value of remission was defined as an HRSD score of 7 or lower,^44^ MADRS score of 9 or lower,^45^ BDI score of 13 or lower^46^ and PHQ-9 score of 9 or lower.^47^
- Family functioning was measured using standardised, validated and reliable measures such as: the Family Assessment Device (FAD),^48^ which was preferentially adopted; (2) the Five-Minute Speech Sample (FMSS), to measure EE;^49^ the Family Attitude Scale (FAS), to measure EE;^50^ the Family Cohesion and Adaptability Evaluation Scale (FACES);^51^ and the Family Environment Scale (FES).^52^

We defined each secondary outcome as follows.

- Patients’ general functioning was assessed using standard measures of general functioning such as: the Global Assessment of Functioning (GAF) scale,^53^ which was preferentially adopted; and the Mental Component Summary (MCS) of the Medical Outcomes Study's (MOS) 36-item Short-Form Health Survey (SF-36).^54^
- Family members’ distress was measured using standardised, validated and reliable measures such as: the BDI,^42^ which was preferentially adopted; the Symptom Checklist (SCL);^55^ and the Kessler Psychological Distress Scale (K6).^56^
- Family members’ quality of life was measured on standardised, validated and reliable measures, such as: the World Health Organization's Well-Being Index (WHO-5),^57^ which was preferentially adopted; and the MCS of the MOS SF-36.^54^
- Drop-out rate from intervention and assessment.

### Data extraction and quality evaluation

The following data were extracted from each study: (a) the first author's name, publication year and the country in which the study was carried out; (b) depression inclusion criteria; (c) sample characteristics; (d) intervention characteristics; number of patients with MDD at baseline; (e) outcome measures; (f) follow-up duration; and (g) number dropping out of assessment. For missing data, attempts were made to contact the principal investigators of the included studies to obtain unreported data or additional details, where possible. Two independent reviewers (F.K. and A.Y.) first separately, and then together, assessed the risk of bias. For RCTs included in the search, the risk of bias was assessed according to the Cochrane risk of bias assessment tool.^58^ The following processes were used to assess bias risk: random sequence generation; allocation concealment; participant masking; masking of outcome assessment; incomplete outcome data; selective reporting; and other sources of bias. Disagreements between the review authors over the risk of bias in individual studies were resolved by discussion, with the involvement of a third review author (N.W.) where necessary. Moreover, we used the Grading of Recommendations Assessment, Development and Evaluation (GRADE) approach to assess the quality of the body of evidence.^59^ The GRADE method involves rating the initial quality of evidence for an association as high, followed by downgrading based on five criteria (risk of bias, inconsistency, indirectness, imprecision and publication bias).

### Statistical analysis

We conducted a meta-analysis using Review Manager 5.4 software for Windows and a random effects model, if at least two studies assessing this specific outcome were obtainable. For continuous outcomes, standard mean differences (s.m.d.) with 95% confidence intervals (95% CI) were calculated as the difference in means between groups divided by the pooled standard deviation (s.d.). For dichotomous data, the risk ratio and 95% CIs were calculated. We calculated dichotomous data from the continuous data using the mean, s.d. and number of participants, according to the statistical methods recommended by Cochrane.^60^ We calculated the number of participants above and below the cut-off value of each depression scale. A cut-off value for remission was defined as an HRSD score of 7 or lower,^44^ BDI score of 13 or lower^46^ and PHQ-9 score of 9 or lower.^47^ If the studies had more than one intervention group, the number of participants in the control group was divided by the number of intervention groups to avoid double-counting of participants in control groups.^61^ For studies that did not report data with s.d., we calculated these values from standard errors or 95% CIs. For pooled analyses, we quantified statistical heterogeneity using the *I*^2^ statistic, which describes the percentage of total variation across trials due to heterogeneity rather than sampling error. If sufficient data were available, subgroup analyses were conducted for type of provider system, type of delivery system, severity of depressive symptoms at baseline, comorbidity of depression, support condition of family members in the control group and relationship with family members. As necessary, we performed sensitivity analyses by removing studies one at a time, to evaluate the stability of the result. If at least ten studies were included in a meta-analysis, we assessed publication bias by visual analysis of funnel plots.

## Results

### Identified studies for the meta-analysis

The literature search retrieved 2717 records. After duplicates were removed, we initially identified a total of 1823 records, and 1756 were excluded after reviewing the titles and abstracts. We obtained 67 relevant articles for full-text review, and a further 50 studies were excluded for the following reasons: 2 were not RCTs, 32 did not include eligible patients, 11 did not include eligible interventions, and 5 were ongoing and did not provide any actual data.^62–66^ Thus, out of 17 remaining records, 9 studies^27,28,30–32,67–78^ met the inclusion criteria for qualitative synthesis and 5 studies^72–78^ met the inclusion criteria for the meta-analysis (supplementary Fig. 1)

### Study characteristics and quality evaluation included in the meta-analysis

This meta-analysis included 5 studies^72–78^ totalling 301 patients with MDD and a family member: 175 in the intervention groups and 126 in the control groups. One study consisted of three separate intervention arms and a shared control group.^72^ Participant details and intervention characteristics are shown in Table 1. Participants met the criteria for MDD according to DSM-IV^72,73,75–77^ or ICD-10^74^ and one study defined MDD with a PHQ-9 score of 15 or higher.^78^ Most interventions included providing information on MDD and confirming how participants understood the condition.^72–78^ Two studies used problem-solving,^74–77^ three studies promoted communication among family members,^72,73,75–77^ four studies enhanced strengths and family coping skills^72,74–78^ and multifamily intervention in two studies enhanced social support.^72,75–77^ Four studies described an FPE intervention that included patients.^72–74,78^ The control condition for family members was that they had not received any treatment. Typical patient treatments in both intervention and control groups included medication,^72–78^ supportive psychotherapy^74–77^ or individual or group psychotherapy^72,73^ (Table 1). The quality of the included studies varied (supplementary Fig. 2). Two of five studies reported an adequate sequence generation.^72,75–77^ Two studies reported allocation to conditions by an independent party.^72,75–77^ Two studies reported incomplete outcome data^74–77^ and two studies published protocols and analysed results according to those protocols.^75–78^ All the RCTs had an unclear or high risk of bias regarding participant masking because of the nature of these studies. Following the GRADE methodology, we graded the quality of evidence for the outcomes of each meta-analysis. All outcomes were graded with a very low degree of certainty (supplementary Table 2). As fewer than ten studies were included in each meta-analysis, funnel plots were not analysed.

**Table 1:** Characteristics of the participants and interventions^a^

		Patients					Intervention for family members in a family psychoeducation group							
Study	Country	*n*	Age, years: mean (s.d.)	Women, %	Duration of illness, years: mean (s.d.)	Depressive symptoms at baseline, mean (s.d.)	Intervention contents^b^	Form of intervention	Number of sessions	Session duration	Intervention duration	Intervention for family members in a control group	Intervention for patients in both the intervention and control groups	Length of follow-up
1 Lemmens et al (2009)^72^	Belgium	35	43.9 (8.3)	80	Unclear	26.6 (9.9)^c^	#1, #3, #4, #5, #6	Multifamily	6	90 min	10 weeks	Not mentioned	Specific therapeutic interventions were offered, mostly in a group format and some individually; the therapeutic techniques and interventions within the programme drew on different conceptual therapeutic frameworks such as non-verbal therapy, CBT, systemic therapy and pharmacological treatment	15 months
		25	40.2 (9.1)	64	Unclear	26 (13.5)^c^	#1, #3, #4, #6	Single family	7	60 min	12 weeks			
		23	13.2 (8.4)	69.6	Unclear	27.3 (10.7)^c^								
2 Seikkula et al (2013)^73^	Finland	35	41.2 (11)	NS	3.16 (4.69)	20 (4.4)^d^	#1, #3, #6	Single family	At least 5	Unclear	36 weeks	Not mentioned	All participants received all treatments considered necessary, including antidepressant medication; the treatment-as-usual control group had individual treatment with possible individual or group psychotherapy sessions and other forms of usual treatment, for example psychiatric consultation, medication and hospital admission	24 months
		31	43.45 (11.2)	NS	3.75 (5.3)	19.8 (4.3)^d^								
3 Kumar et al (2015)^74^	India	40	33 (11.63)	40	8.1 (3.77)	24.23 (3.00)^d^	#1, #2, #4, #6	Single family	4	Unclear	8 weeks	Not mentioned	Both groups received routine unstructured counselling.	3 months
		40	39.33 (11.15)	37.5	9 (3.65)	22.48 (4.31)^d^								
4 Katsuki et al (2018)^75,76,77^	Japan	25	43.5 (17.4)	44	7.0 (6.4)	22.2 (10.4)^c^	#1, #2, #3, #4, #5	Multifamily	4	120 min	6 weeks	One 45 min counselling session administered by nurses; one counselling by nurses was in the treatment as usual	Out-patient treatment consisted of evaluation of psychiatric symptoms, antidepressant pharmacotherapy and supportive psychotherapy; in-patient treatment consisted of sufficient rest for the patient, evaluation of psychiatric symptoms, antidepressant pharmacotherapy and supportive psychotherapy	8 months
		24	43.9 (18.2)	54.2	9.2 (8.4)	25.7 (15.1)^c^								
5 Hinton et al (2020)^78^	USA	15	60.2 (9.6)	0	Unclear	14.8 (2.9)^e^	#1, #4, #6	Single family	At least 10^f^	Unclear	Unclear	Standard psychoeducational materials on depression	Control participants received usual care in the clinic augmented by psychoeducation	6 months
		8	57.4 (4.8)	0	Unclear	15.3 (4.1)^e^								
6 Clarkin et al (1990)^30,31,32^	USA	17	38.4 (12.7)	55	Unclear	Unclear	#1, #2, #3, #4, #6	Multifamily	At least 6	14–60 min	Unclear	Standard treatment on depression; questions concerning the hospital or the patient's illness were dealt with according to guidelines that permit information exchange while minimising intervention in the family system	All patients received multimodal hospital treatment, including a full range of diagnostic services and individual, group, milieu, activity and somatic therapies	19 months
		12												
7 Hu Xiong et al (2007)^71^	China	39	36 (6)	64.1	5.6 (5.4)	28.71 (7.62)^d^	Unclear	Unclear	Unclear	Unclear	Unclear	Family members were given standard treatment on depression	Not mentioned	24 months
		37	35 (6)	64.8	5.9 (5.8)	28.95 (7.46)^d^								
8 Fiorillo et al (2011)^28,67^	Italy	22	48.6 (10.8)	59	Unclear	Unclear	#1, #2, #3	Single family	12	90 min	Unclear	Family members received treatment as usual plus an informative brief intervention	Not mentioned	6 months
		22												
9 Shimazu et al (2011)^27,68,69,70^	Japan	24	59.2 (14.6)	37.5	11.6 (27)	13.4 (8.3)^d^	#1, #2,#3, #4, #5	Multifamily	4	90–120 min for 2 weeks	6 weeks	Not mentioned	Out-patient treatment as usual consisted of evaluation of psychiatric symptoms, assessment and management of drug treatment and supportive psychotherapy once a fortnight	9 months
		30	60.9 (13.0)	50	11.0 (2.0)	13.7 (10.5)^d^								

CBT, cognitive–behavioural therapy.

a. For each study, the upper row of data (the upper two rows for study 1) relates to the intervention group and the lower row to the control group.

b. #1: providing information on the illness (major depressive disorder) and ensuring that people have an understanding of the illness. #2: using problem-solving. #3: enhancement of communication among family members. #4: enhancement of strength and coping. #5: enhancement of social supports. #6: including the patient.

c. Beck Depression Inventory-II.

d. Hamilton Rating Scale for Depression.

e. Patient Health Questionnaire-9.

f. Patient alone and/or with family members.

### Effects on patients’ depressive symptoms

**Fig. 1:**
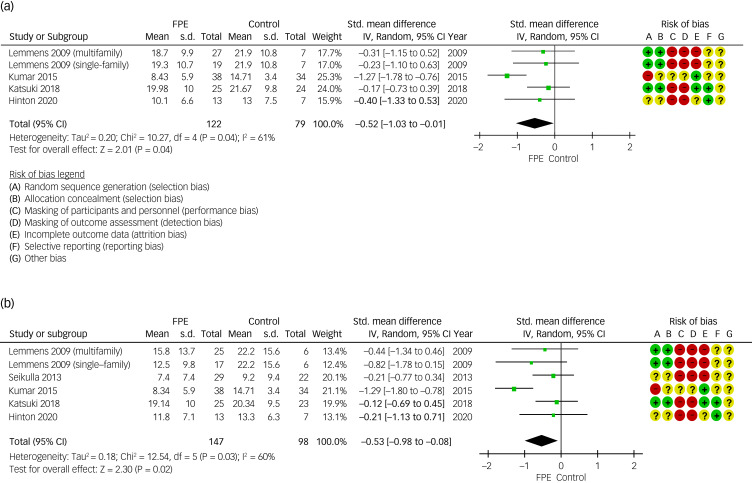
Forest plots for severity of patients’ depressive symptoms. IV, inverse variance; FPE, family psychoeducation.

**Fig. 2:**
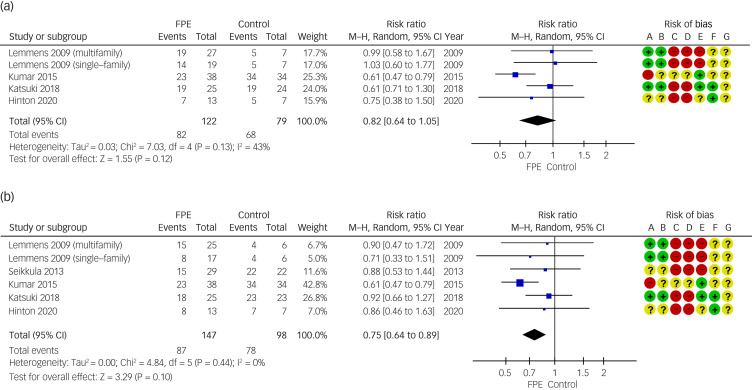
Forest plots for depression above the threshold. M–H, Mantel–Haenszel; FPE, family psychoeducation.

The effect of FPE on patients’ depressive symptom severity as primary outcomes, compared with the control condition, at 16 weeks (from 12 weeks to 20 weeks) was available for five comparisons of four RCTs,^72,74–78^ and final follow-up data were available for six comparisons of five RCTs.^72–78^ The meta-analysis of combined data showed a significant improvement in patients’ depressive symptoms at 16 weeks (s.m.d. = −0.52; 95% CI 1.03 to −0.01) and at a final follow-up (s.m.d. = −0.53; 95% CI −0.98 to −0.08). Fig. 1 illustrates forest plots of the standard mean differences and their confidence intervals. Heterogeneity was substantial at 16 weeks (*I*^2^ = 61%) and at a final follow-up (*I*^2^ = 60%). Although two studies used clinician-rated instruments (HRSD)^73,74^ for depressive symptom assessment, three used self-report instruments (BDI, PHQ-9)^72,75–78^ (Table 1). The final follow-up period ranged from 3 to 24 months (Table 1). Fig. 2 shows the results of a meta-analysis for depression above the threshold in the same population. The meta-analysis of the depression rate above threshold using the pooled relative risks showed a non-significant improvement rate at 16 weeks (risk ratio 0.82; 95% CI 0.64–1.05) and a significant improvement rate at final follow-up (risk ratio 0.75; 95% CI 0.64–0.89). Heterogeneity was moderate at 16 weeks (*I*^2^ = 43%) and low at final follow-up (*I*^2^ = 0%).

### Family functioning

The meta-analysis on the effect of FPE on family functioning as one of the primary outcomes, using the FAS, showed a non-significant improvement at both 16 weeks (m.d. = 4.15; 95% CI −11.66 to 19.96) and final follow-up (m.d. = 7.50; 95% CI −7.62 to 22.62; supplementary Fig. 3). Only one study^75–77^ reported family functioning and family members’ distress.

### Other comparisons

Data on The effect of FPE on patients’ general functioning, compared with the control condition, at 16 weeks were available in two RCTs^74–77^ and at a final follow-up in three RCTs.^73–77^ The meta-analysis of the combined data showed a non-significant improvement in patients’ general functioning using the MSC of SF-36 or the GAF at 16 weeks (s.m.d. = 0.64; 95% CI −0.15 to 1.44) and at a final follow-up (s.m.d. = 0.39; 95% CI −0.28 to 1.06; supplementary Fig. 4). There was no significant effect of FPE on family members’ distress using the BDI at 16 weeks (m.d. = −1.85; 95% CI −7.12 to 3.42) or at a final follow-up (m.d. = 0.95; 95% CI −4.60 to 6.50; supplementary Fig. 5). We calculated the risk ratio of the drop-out rate from intervention and assessment (supplementary Fig. 6). In terms of the drop-out rate from intervention, four^72,74–78^ of five studies had no one dropping out from the intervention or control group. Considering the drop-out rate from assessment, one^75–77^ out of five studies had no one dropping out from the intervention or control group. These studies were excluded from the meta-analysis. There was no significant difference in the drop-out rate between the intervention and control groups (risk ratio 0.59; 95% CI 0.24–1.47), but a significant difference was observed in the drop-out rate from assessment (risk ratio 0.50; 95% CI 0.31–0.80).

### Subgroup and sensitivity analysis

The small number of studies in the meta-analysis did not allow for subgroup analysis. Sensitivity analysis was not performed because there were no outlier data.

### Studies excluded from the meta-analysis

Four studies^27,28,30–32,67–71^ of ten records had no data available for our meta-analysis. Three studies^27,28,30–32,67–70^ did not include adequate means and standard deviations. One study^71^ did not provide detailed contents of the FPE intervention. We requested the detailed data from the respective authors but could not retrieve them, as they were already discarded or the authors could not be reached. Therefore, these studies could not be included in the meta-analysis. These studies’ participants met the criteria for MDD according to DSM-III,^30–32^ DSM-IV^27,28,68–70^ or the Chinese Classification of Mental Disorders Version 3.^71^ Interventions for family members are shown in Table 1. The Clarkin study^30–32^ reported no significant improvement (*F* = 3.12, *P* = 0.09 at 18 months) in the patients' general functioning, measured on the Global Assessment Scale. Hu Xiong et al^71^ reported significant improvement (*P* < 0.01 at 24 months) in patients’ depressive symptoms, measured on the HRSD. Fiorillo et al's study^28,67^ reported a significant reduction in patients’ symptom severity (*P* < 0.05) and family burden (*P* < 0.05) compared with the control group. Shimazu et al^27,68–70^ reported that patients who had achieved full or partial remission from an acute depressive episode had a significantly lower relapse rate during a 9-month follow-up period compared with those in the control group (risk ratio 0.17; 95% CI 0.04–0.66; number needed to treat 2.4; 95% CI 1.6–4.9).

## Discussion

To the best of our knowledge, this is the first meta-analytical study exploring the effectiveness of FPE for MDD. We found that FPE had a small but statistically significant effect on the depressive symptoms of people with MDD, in both the short and long term. This suggests that FPE as a psychosocial intervention may be expected to improve the depressive symptoms of people with MDD. Major depression has many effects on family functioning.^79^ Individuals with a neurobiological vulnerability to major mental disorders are highly sensitive to stressors. Coping with stressors can be impeded by ineffective efforts at communication and problem-solving among family members. Expressing ideas and feelings, making requests of each other and dividing big problems into small steps are skills that few families have mastered.^39^ In a survey of mental health workers who support people with depression and their caregivers, 80% of participants answered yes to the question of whether the family members of a person with depression could prevent recurrence by gaining appropriate knowledge about the condition.^80^ However, only 23% of participants answered yes to the question regarding whether the caregivers were learning to manage depression appropriately. The FPE intervention may help families obtain knowledge about MDD, develop coping strategies for problems in daily life and improve communication between family members and patients. Appropriate coping strategies among family members, such as problem-solving and positive thinking, may reduce the stress for both the patients and family members.^9^ This may also reduce caregivers' EE and have a positive effect on the patients’ prognosis.^13–15^ This is believed to result in reduced stress for both family members and patients and reduced depressive symptoms among patients. However, although the EE-lowering effect of FPE has been confirmed for schizophrenia,^11^ it has not yet been confirmed for MDD. In addition, there was no significant improvement in family functioning and family members’ distress in the present study. Thus far, only a few studies have examined the effects of family functioning and family members’ distress on people with MDD. Additionally, the studies included in the present study differed from each other in terms of intervention contents and the usual treatment type for patients in the intervention and control groups (Table 1); the quality of most studies in this field was suboptimal. Furthermore, four studies^72–74,78^ used an FPE intervention including patients whereas one study ^75–77^ was with a family member only. Because of the small number of studies, it was not possible to analyse each intervention and its control conditions separately using subgroup analyses. Therefore, these results should be considered with caution and verified through further research.

Although there are several meta-analyses^81,82^ on the effect of psychoeducation on people with MDD, to date, there has been no meta-analysis on FPE for MDD. However, there are two narrative reviews on family therapy for MDD, of which one involved FPE^33^ and one comprised both family therapy and FPE.^18^ The single systematic review^33^ of FPE for MDD included six RCTs and three single-arm trials. This review stated that the results provide preliminary evidence that FPE leads to improved outcomes for patients’ functioning and family caregivers’ well-being when caring for people with depression. Another review^18^ involved six RCTs and addressed not only FPE, but also other types of family therapy. This review revealed that despite the lack of high-quality evidence in the field, family therapy is already a widely used intervention for depression. Our findings, however, are the first to show quantitative results. Additionally, four studies^27,28,30–32,67–71^ identified during the present study that were not included in the meta-analysis report a significant effect of FPE on patients’ depressive symptoms or general functioning. In particular, Shimazu et al reported high prevention of recurrence of MDD when applying FPE.^27,68–70^ Moreover, the FPE intervention in that study targeted family members only. Overall, family care is important for MDD treatment, and FPE can be expected to be effective in those recovering from MDD.

### Limitations of the study

This meta-analysis had several limitations. First, the sample size across the included studies was relatively small. A meta-analysis on such a small sample limits any inference of a pooled effect. Second, only five RCTs were included in the meta-analysis and all of the included studies were of low quality (supplementary Fig. 2). In several studies it was unclear whether the authors concealed random allocation. Almost all the studies did not mask the participants and outcome assessors. Third, according to the GRADE framework, the certainty of each outcome was very low (supplementary Table 2). The total number of participants in this meta-analysis was small and the *I*^2^ value measuring inconsistency indicated substantial heterogeneity. Some outcomes showed low risk of indirectness, as each study had a different follow-up period. Fourth, several questionnaires that measured depressive symptoms included self-report questionnaires. Fifth, in the meta-analysis of the depression rate above threshold, when we converted continuous data to dichotomous data, we did not examine whether the normal distribution held true. Last, in the studies included in the present study, participants with MDD received additional treatments in both the intervention and control groups. Considering these limitations, the results regarding the effectiveness of FPE for MDD may change in future studies. However, the strengths of this study were that all the steps were performed by two or more independent researchers and a wide range of literature databases were included, such as ProQuest Dissertations and Theses.

### Implications

As one of the psychosocial interventions, FPE can be expected to improve depressive symptoms in people with MDD. These results, however, should be confirmed in further randomised trials because of the clinical heterogeneity and the low quality of the included studies and because the clinician-rated outcomes were not significant. Higher-quality RCTs that include large samples and masking of assessors are needed. Researchers must publish study protocols prior to research and subsequently analyse data according to the respective protocols. The effectiveness of FPE for MDD is yet to be validated and it is not yet highly recommended. The results of the present study suggest that FPE for MDD can be expected to promote symptom-improving effects and have a recurrence-prevention effect when combined with other treatments.

## Data Availability

The data-sets used and/or analysed during the current study are available from the corresponding author on reasonable request.

## Supplementary material

For supplementary material accompanying this paper visit http://doi.org/10.1192/bjo.2022.543. click here to view supplementary material
