# Supplementary material for: Effectiveness of family psychoeducation for major depressive disorder: systematic review and meta-analysis
Source: BJPsych Open. 2022 Aug 2;8(5):e148. doi: 10.1192/bjo.2022.543 (PMC9380172; doi:10.1192/bjo.2022.543)
Supplement: Supplementary file 1 [file S2056472422005439sup001.zip › Supplementary Table 2.pdf]

Supplementary Table 2. Summary of evidence quality based on the GRADE approach

Certainty assessment							№ of patients		Effect		Certainty	Importance
№ of studies	Study design	Risk of bias	Inconsistency	Indirectness	Imprecision	Other considerations	Depressive symptoms	placebo	Relative (95% CI)	Absolute (95% CI)		
**Severity of depressive symptoms of patients at 16 weeks**												
5	randomised trials	serious	serious	not serious	serious	none	122	79	-	SMD **0.52 lower** (1.03 lower to 0.01 lower)	⨁◯◯◯ Very low	8
**Severity of depressive symptoms of patients at last follow-up**												
6	randomised trials	serious	serious	serious	serious	none	147	98	-	SMD **0.53 lower** (0.98 lower to 0.08 lower)	⨁◯◯◯ Very low	9
**Depression above threshold at 16 weeks**												
5	randomised trials	serious	serious	not serious	serious	none	82/122 (67.2%)	68/79 (86.1%)	**RR 0.82** (0.64 to 1.05)	**155 fewer per 1,000** (from 310 fewer to 43 more)	⨁◯◯◯ Very low	8
**Depression above threshold at last follow-up**												
6	randomised trials	serious	not serious	serious	serious	none	87/147 (59.2%)	78/98 (79.6%)	**RR 0.75** (0.64 to 0.89)	**199 fewer per 1,000** (from 287 fewer to 88 fewer)	⨁◯◯◯ Very low	9
**Family functioning at 16 weeks**												
1	randomised trials	serious	not serious	not serious	very serious	none	25	23	-	MD **4.15 higher** (11.66 lower to 19.96 higher)	⨁◯◯◯ Very low	6
**Family functioning at last follow-up**												
1	randomised trials	serious	not serious	not serious	very serious	none	25	23	-	MD **7.5 higher** (7.62 lower to 22.62 higher)	⨁◯◯◯ Very low	7

CI: confidence interval; **MD:** mean difference; **RR:** risk ratio; **SMD:** standardised mean difference

Certainty assessment							№ of patients		Effect		Certainty	Importance
№ of studies	Study design	Risk of bias	Inconsistency	Indirectness	Imprecision	Other considerations	Depressive symptoms	placebo	Relative (95% CI)	Absolute (95% CI)		
**Patients' general functioning at 16 weeks**												
2	randomised trials	serious	very serious	not serious	serious	none	63	58	-	SMD **0.64 higher** (0.15 lower to 1.44 higher)	⨁◯◯◯ Very low	3
**Patients' general functioning at last follow-up**												
3	randomised trials	serious	very serious	serious	serious	none	92	79	-	SMD **0.39 higher** (0.28 lower to 1.06 higher)	⨁◯◯◯ Very low	4
**Family members' distress at 16 weeks**												
1	randomised trials	serious	not serious	not serious	very serious	none	25	23	-	MD **1.85 lower** (7.12 lower to 3.42 higher)	⨁◯◯◯ Very low	2
**Family members' distress at last follow up**												
1	randomised trials	serious	not serious	not serious	very serious	none	25	23	-	MD **0.95 higher** (4.6 lower to 6.5 higher)	⨁◯◯◯ Very low	2
**Drop-out rate from intervention**												
1	randomised trials	serious	not serious	not serious	very serious	none	6/35 (17.1%)	9/31 (29.0%)	**RR 0.59** (0.24 to 1.47)	**119 fewer per 1,000** (from 221 fewer to 136 more)	⨁◯◯◯ Very low	2
**Drop-out rate from assessment**												
5	randomised trials	serious	not serious	serious	serious	none	24/150 (16.0%)	28/103 (27.2%)	**RR 0.50** (0.31 to 0.80)	**136 fewer per 1,000** (from 188 fewer to 54 fewer)	⨁◯◯◯ Very low	4

CI: confidence interval; **MD:** mean difference; **RR:** risk ratio; **SMD:** standardised mean difference
