# Supplementary material for: Effectiveness of family psychoeducation for major depressive disorder: systematic review and meta-analysis
Source: BJPsych Open. 2022 Aug 2;8(5):e148. doi: 10.1192/bjo.2022.543 (PMC9380172; doi:10.1192/bjo.2022.543)
Supplement: Supplementary file 1 [file S2056472422005439sup001.zip › Supplementary Table 1.pdf]

Supplementary Table 1. The search terms.

PubMed

http://www.ncbi.nlm.nih.gov/pubmed?otool=leiden

https://pmlegacy.ncbi.nlm.nih.gov/

(("family education"[tiab] OR "family psychoeducation"[tiab] OR "family psycho education"[tiab] OR (("Family"[mesh] OR "Family Therapy"[mesh] OR "Family Relations"[mesh] OR "Family Health"[mesh]) AND ("Health Education"[mesh] OR "Patient Education as Topic"[mesh])) OR (("psychoeducat*"[tiab] OR "psycho educat*"[tiab]) AND ("family"[tiab] OR "families"[tiab] OR "Family"[mesh] OR "Family Therapy"[mesh] OR "Family Relations"[mesh] OR "Family Health"[mesh])) OR "Family Therapy"[mesh] OR "family therap*"[tiab] OR "family intervention*"[tiab]) AND ("Depressive Disorder"[Mesh] OR "Depressive Disorder*"[tiab] OR "Depression"[tiab]) AND ("randomized controlled trial"[pt] OR "controlled clinical trial"[pt] OR "randomized"[tiab] OR "placebo"[tiab] OR "clinical trials as topic"[mesh:noexp] OR "randomly"[tiab] OR "trial"[ti]) NOT ("Animals"[mesh] NOT "Humans"[mesh]))

MEDLINE via OVID

http://gateway.ovid.com/ovidweb.cgi?T=JS&MODE=ovid&NEWS=n&PAGE=main&D=medall

(("family education".ti,ab OR "family psychoeducation".ti,ab OR "family psycho education".ti,ab OR ((exp "Family"/ OR exp "Family Therapy"/ OR exp "Family Relations"/ OR exp "Family Health"/) AND (exp "Health Education"/ OR exp "Patient Education as Topic"/)) OR (("psychoeducat*".ti,ab OR "psycho educat*".ti,ab) AND ("family".ti,ab OR "families".ti,ab OR exp "Family"/ OR exp "Family Therapy"/ OR exp "Family Relations"/ OR exp "Family Health"/)) OR "exp Family Therapy"/ OR "family therap*".ti,ab OR "family intervention*".ti,ab) AND (exp "Depressive Disorder"/ OR "Depressive Disorder*".ti,ab OR "Depression".ti,ab) AND (exp "randomized controlled trial"/ OR exp "controlled clinical trial"/ OR "randomized".ti,ab OR "placebo".ti,ab OR "clinical trials as topic"/ OR "randomly".ti,ab OR "trial".ti) NOT (exp "Animals"/ NOT exp "Humans"/))

Embase

http://ovidsp.ovid.com/ovidweb.cgi?T=JS&PAGE=main&MODE=ovid&D=oemezd

(("family education".ti,ab OR "family psychoeducation".ti,ab OR "family psycho education".ti,ab OR ((exp *"Family"/ OR exp *"Family Therapy"/ OR exp *"Family Relation"/ OR *"Family Health"/) AND (exp *"Health Education"/ OR exp *"Patient Education"/)) OR (("psychoeducation"/ OR "psychoeducat*".ti,ab OR

"psycho educat*".ti,ab) AND ("family".ti,ab OR "families".ti,ab OR exp *"Family"/ OR exp *"Family Therapy"/ OR exp *"Family Relation"/ OR *"Family Health"/)) OR *"Family Therapy"/ OR "family therap*".ti,ab OR "family intervention*".ti,ab) AND (*"Major Depression"/ OR "Depressive Disorder*".ti,ab OR "Depression".ti,ab) AND ("crossover procedure"/ OR "double-blind procedure"/ OR "randomized controlled trial"/ OR "single-blind procedure"/ OR (random* OR factorial* OR crossover* OR (cross ADJ2 over*) OR placebo* OR (doubl* ADJ2 blind*) OR (singl* ADJ2 blind*) OR assign* OR allocat* OR volunteer*).mp) NOT (exp "Animals"/ NOT exp "Humans"/))

Web of Science

http://isiknowledge.com/wos

(ti=("family education" OR "family psychoeducation" OR "family psycho education" OR (("Family" OR "Family Therapy" OR "Family Relation" OR "Family Health") AND ("Health Education" OR "Patient Education")) OR (("psychoeducation" OR "psychoeducat*" OR "psycho educat*") AND ("family" OR "families" OR "Family" OR "Family Therapy" OR "Family Relation" OR "Family Health")) OR "Family Therapy" OR "family therap*" OR "family intervention*") AND ts=("Major Depression" OR "Depressive Disorder*" OR "Depression") AND ts=("crossover procedure" OR "double-blind procedure" OR "randomized controlled trial" OR "single-blind procedure" OR (random* OR factorial* OR crossover* OR (cross NEAR3 over*) OR placebo* OR (doubl* NEAR3 blind*) OR (singl* NEAR3 blind*) OR assign* OR allocat* OR volunteer*)) NOT ti=("veterinary" OR "rabbit" OR "rabbits" OR "animal" OR "animals" OR "mouse" OR "mice" OR "rodent" OR "rodents" OR "rat" OR "rats" OR "pig" OR "pigs" OR "porcine" OR "horse" OR "horses" OR "equine" OR "cow" OR "cows" OR "bovine" OR "goat" OR "goats" OR "sheep" OR "ovine" OR "canine" OR "dog" OR "dogs" OR "feline" OR "cat" OR "cats")) **OR** (ts=("family education" OR "family psychoeducation" OR "family psycho education" OR (("Family" OR "Family Therapy" OR "Family Relation" OR "Family Health") AND ("Health Education" OR "Patient Education")) OR (("psychoeducation" OR "psychoeducat*" OR "psycho educat*") AND ("family" OR "families" OR "Family" OR "Family Therapy" OR "Family Relation" OR "Family Health")) OR "Family Therapy" OR "family therap*" OR "family intervention*") AND ti=("Major Depression" OR "Depressive Disorder*" OR "Depression") AND ts=("crossover procedure" OR "double-blind procedure" OR "randomized controlled trial" OR "single-blind procedure" OR (random* OR factorial* OR crossover* OR (cross NEAR3 over*) OR placebo* OR (doubl* NEAR3 blind*) OR (singl* NEAR3 blind*) OR assign* OR allocat* OR volunteer*)) NOT ti=("veterinary" OR "rabbit" OR "rabbits" OR "animal" OR "animals" OR "mouse" OR "mice" OR "rodent" OR "rodents" OR "rat" OR "rats" OR "pig" OR "pigs" OR "porcine" OR "horse" OR "horses" OR "equine" OR "cow" OR "cows" OR "bovine" OR "goat" OR "goats" OR "sheep" OR "ovine" OR "canine" OR "dog" OR "dogs" OR "feline" OR "cat" OR "cats"))

Cochrane
https://www.cochranelibrary.com/advanced-search/search-manager

((("family education" OR "family psychoeducation" OR "family psycho education"):ti,ab,kw OR (("Family" OR "Family Therapy" OR "Family Relation" OR "Family Health") AND ("Health Education" OR "Patient Education")):ti OR (("psychoeducation" OR "psychoeducat*" OR "psycho educat*") AND ("family" OR "families" OR "Family" OR "Family Therapy" OR "Family Relation" OR "Family Health")):ti OR ("Family Therapy" OR "family therap*" OR "family intervention*"):ti) AND ("Major Depression" OR "Depressive Disorder*" OR "Depression"):ti,ab,kw)

Emcare http://ovidsp.ovid.com/ovidweb.cgi?T=JS&NEWS=n&CSC=Y&PAGE=main&D=emcr

(("family education".ti,ab OR "family psychoeducation".ti,ab OR "family psycho education".ti,ab OR ((exp *"Family"/ OR exp *"Family Therapy"/ OR exp *"Family Relation"/ OR *"Family Health"/) AND (exp *"Health Education"/ OR exp *"Patient Education"/)) OR (("psychoeducation"/ OR "psychoeducat*".ti,ab OR "psycho educat*".ti,ab) AND ("family".ti,ab OR "families".ti,ab OR exp *"Family"/ OR exp *"Family Therapy"/ OR exp *"Family Relation"/ OR *"Family Health"/)) OR *"Family Therapy"/ OR "family therap*".ti,ab OR "family intervention*".ti,ab) AND (*"Major Depression"/ OR "Depressive Disorder*".ti,ab OR "Depression".ti,ab) AND ("crossover procedure"/ OR "double-blind procedure"/ OR "randomized controlled trial"/ OR "single-blind procedure"/ OR (random* OR factorial* OR crossover* OR (cross ADJ2 over*) OR placebo* OR (doubl* ADJ2 blind*) OR (singl* ADJ2 blind*) OR assign* OR allocat* OR volunteer*).mp) NOT (exp "Animals"/ NOT exp "Humans"/))

PsycINFO
http://search.ebscohost.com/login.aspx?authtype=ip,uid&profile=lumc&defaultdb=psyh

(DE(("family education" OR "family psychoeducation" OR "family psycho education" OR (("Family" OR "Family Therapy" OR "Family Relation" OR "Family Health") AND ("Health Education" OR "Patient Education")) OR (("psychoeducation" OR "psychoeducat*" OR "psycho educat*") AND ("family" OR "families" OR "Family" OR "Family Therapy" OR "Family Relation" OR "Family Health")) OR "Family Therapy" OR "family therap*" OR "family intervention*") AND ("Major Depression" OR "Depressive Disorder*" OR "Depression" OR "Major Depression" OR "Anaclitic Depression" OR "Dysthymic Disorder" OR "Endogenous Depression" OR "Late Life Depression" OR "Postpartum Depression" OR "Reactive Depression" OR "Recurrent Depression" OR "Treatment Resistant Depression")) **OR** TI(("family education" OR "family psychoeducation" OR "family psycho education" OR (("Family" OR "Family Therapy" OR "Family Relation" OR "Family Health") AND ("Health Education" OR "Patient Education")) OR (("psychoeducation"

OR "psychoeducat*" OR "psycho educat*") AND ("family" OR "families" OR "Family" OR "Family Therapy" OR "Family Relation" OR "Family Health")) OR "Family Therapy" OR "family therap*" OR "family intervention*") AND ("Major Depression" OR "Depressive Disorder*" OR "Depression" OR "Major Depression" OR "Anaclitic Depression" OR "Dysthymic Disorder" OR "Endogenous Depression" OR "Late Life Depression" OR "Postpartum Depression" OR "Reactive Depression" OR "Recurrent Depression" OR "Treatment Resistant Depression")) **OR** SU(("family education" OR "family psychoeducation" OR "family psycho education" OR (("Family" OR "Family Therapy" OR "Family Relation" OR "Family Health") AND ("Health Education" OR "Patient Education")) OR (("psychoeducation" OR "psychoeducat*" OR "psycho educat*") AND ("family" OR "families" OR "Family" OR "Family Therapy" OR "Family Relation" OR "Family Health")) OR "Family Therapy" OR "family therap*" OR "family intervention*") AND ("Major Depression" OR "Depressive Disorder*" OR "Depression" OR "Major Depression" OR "Anaclitic Depression" OR "Dysthymic Disorder" OR "Endogenous Depression" OR "Late Life Depression" OR "Postpartum Depression" OR "Reactive Depression" OR "Recurrent Depression" OR "Treatment Resistant Depression")) **OR** AB(("family education" OR "family psychoeducation" OR "family psycho education" OR (("Family" OR "Family Therapy" OR "Family Relation" OR "Family Health") AND ("Health Education" OR "Patient Education")) OR (("psychoeducation" OR "psychoeducat*" OR "psycho educat*") AND ("family" OR "families" OR "Family" OR "Family Therapy" OR "Family Relation" OR "Family Health")) OR "Family Therapy" OR "family therap*" OR "family intervention*") AND ("Major Depression" OR "Depressive Disorder*" OR "Depression" OR "Major Depression" OR "Anaclitic Depression" OR "Dysthymic Disorder" OR "Endogenous Depression" OR "Late Life Depression" OR "Postpartum Depression" OR "Reactive Depression" OR "Recurrent Depression" OR "Treatment Resistant Depression")))

(SU("Treatment Effectiveness Evaluation") OR DE("Treatment Effectiveness Evaluation") OR DE "Treatment Outcomes" OR DE "Psychotherapeutic Outcomes" OR DE "Side Effects (Treatment)" OR DE "Treatment Compliance" OR DE "Treatment Duration" OR DE "Treatment Refusal" OR DE "Treatment Termination" OR DE "Treatment Withholding" OR SU "Treatment Outcomes" OR SU "Psychotherapeutic Outcomes" OR SU "SiSU Effects (Treatment)" OR SU "Treatment Compliance" OR SU "Treatment Duration" OR SU "Treatment Refusal" OR SU "Treatment Termination" OR SU "Treatment Withholding" OR SU("Placebo") OR SU("Followup Studies") OR DE("Placebo") OR DE("Followup Studies") OR TX(placebo* OR random* OR RCT OR "comparative stud*" OR (clinical N3 trial*) OR (research N3 design) OR (evaluat* N3 stud*) OR (prospectiv* N3 stud*) OR ((singl* OR doubl* OR trebl* OR tripl*) N3 (blind* OR mask*))) OR DE "Randomized Controlled Trials" OR DE "Randomized Clinical Trials" OR DE "Randomized Clinical Trials")

ProQuest Dissertation and Theses Solutions
https://databases.library.leiden.edu/redirect/990027618100302711

NOFT(("family education" OR "family psychoeducation" OR "family psycho education" OR (("Family" OR

"Family Therapy" OR "Family Relation" OR "Family Health") AND ("Health Education" OR "Patient Education")) OR (("psychoeducation" OR "psychoeducat*" OR "psycho educat*") AND ("family" OR "families" OR "Family" OR "Family Therapy" OR "Family Relation" OR "Family Health")) OR "Family Therapy" OR "family therap*" OR "family intervention*") AND ("Major Depression" OR "Depressive Disorder*" OR "Depression" OR "Major Depression" OR "Anaclitic Depression" OR "Dysthymic Disorder" OR "Endogenous Depression" OR "Late Life Depression" OR "Postpartum Depression" OR "Reactive Depression" OR "Recurrent Depression" OR "Treatment Resistant Depression")) AND

NOFT(RCT* OR random* OR ("Treatment Effectiveness Evaluation") OR ("Treatment Effectiveness Evaluation") OR "Treatment Outcomes" OR "Psychotherapeutic Outcomes" OR "Side Effects (Treatment)" OR "Treatment Compliance" OR "Treatment Duration" OR "Treatment Refusal" OR "Treatment Termination" OR "Treatment Withholding" OR "Treatment Outcomes" OR "Psychotherapeutic Outcomes" OR "SiSU Effects (Treatment)" OR "Treatment Compliance" OR "Treatment Duration" OR "Treatment Refusal" OR "Treatment Termination" OR "Treatment Withholding" OR ("Placebo") OR ("Followup Studies") OR ("Placebo") OR ("Followup Studies") OR (placebo* OR random* OR RCT OR "comparative stud*" OR (clinical N3 trial*) OR (research N3 design) OR (evaluat* N3 stud*) OR (prospectiv* NEAR/3 stud*) OR ((singl* OR doubl* OR trebl* OR tripl*) N3 (blind* OR mask*))) OR "Randomized Controlled Trials" OR "Randomized Clinical Trials" OR "Randomized Clinical Trials")
