# Supplementary material for: Effectiveness of family psychoeducation for major depressive disorder: systematic review and meta-analysis
Source: BJPsych Open. 2022 Aug 2;8(5):e148. doi: 10.1192/bjo.2022.543 (PMC9380172; doi:10.1192/bjo.2022.543)

Supplementary Figure 2. Risk of bias assessment summary

(a)

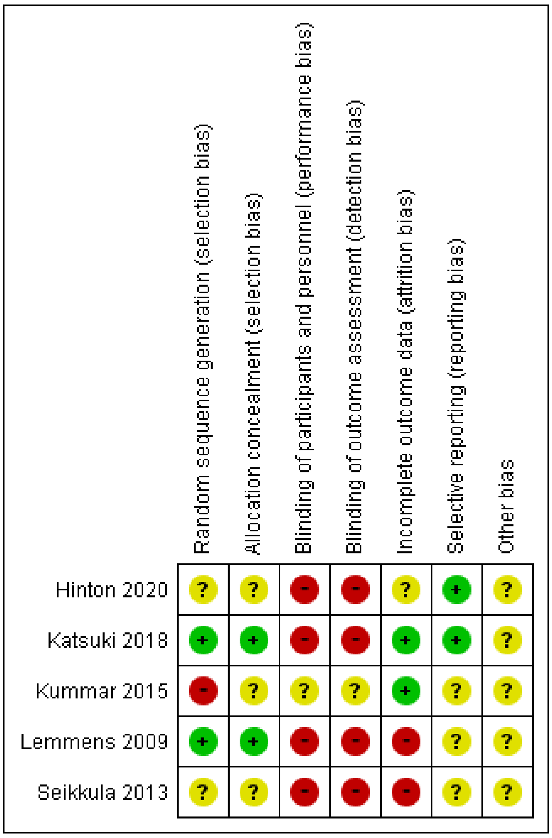

(b)

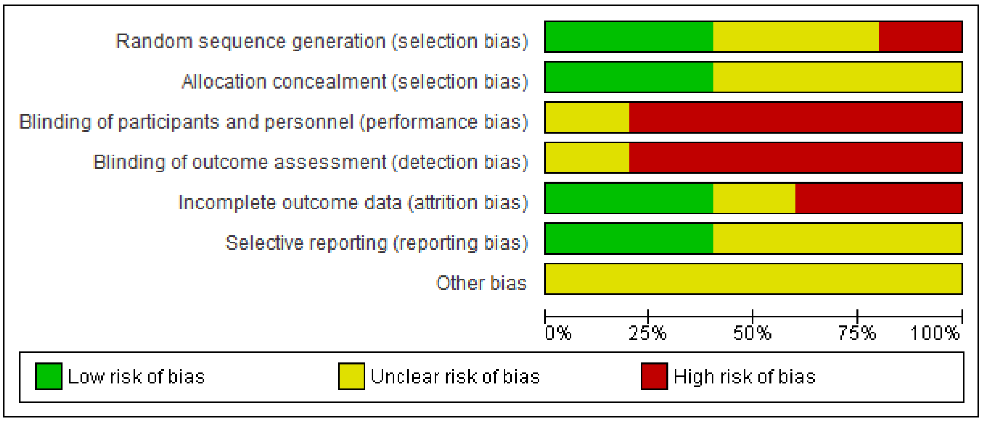

Supplement: Supplementary file 1 [file S2056472422005439sup001.zip › Supplementary Figure 2.pdf]
