# Supplementary material for: Effectiveness of family psychoeducation for major depressive disorder: systematic review and meta-analysis
Source: BJPsych Open. 2022 Aug 2;8(5):e148. doi: 10.1192/bjo.2022.543 (PMC9380172; doi:10.1192/bjo.2022.543)
Supplement: Supplementary file 1 [file S2056472422005439sup001.zip › Supplementary Fig3-6.pdf]

(a)

	FPE			Control				Mean Difference	
Study or Subgroup	Mean	SD	Total	Mean	SD	Total	Weight	IV, Random, 95% CI	Year
Katsuki 2018	41.33	29.5	25	37.18	26.3771	23	100.0%	4.15 [-11.66, 19.96]	2018
**Total (95% CI)**			**25**			**23**	**100.0%**	**4.15 [-11.66, 19.96]**	

Heterogeneity: Not applicable
Test for overall effect: Z = 0.51 (P = 0.61)

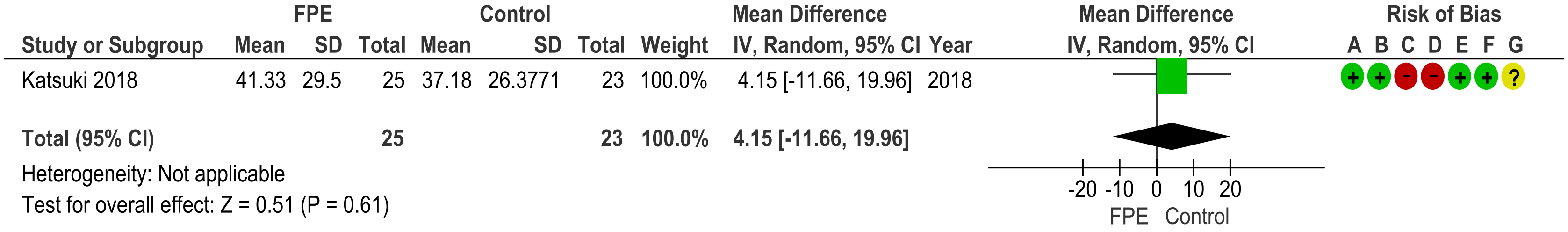

(b)

	FPE			Control				Mean Difference	
Study or Subgroup	Mean	SD	Total	Mean	SD	Total	Weight	IV, Random, 95% CI	Year
Katsuki 2018	39.86	28.5	25	32.36	24.9383	23	100.0%	7.50 [-7.62, 22.62]	2018
**Total (95% CI)**			**25**			**23**	**100.0%**	**7.50 [-7.62, 22.62]**	

Heterogeneity: Not applicable
Test for overall effect: Z = 0.97 (P = 0.33)

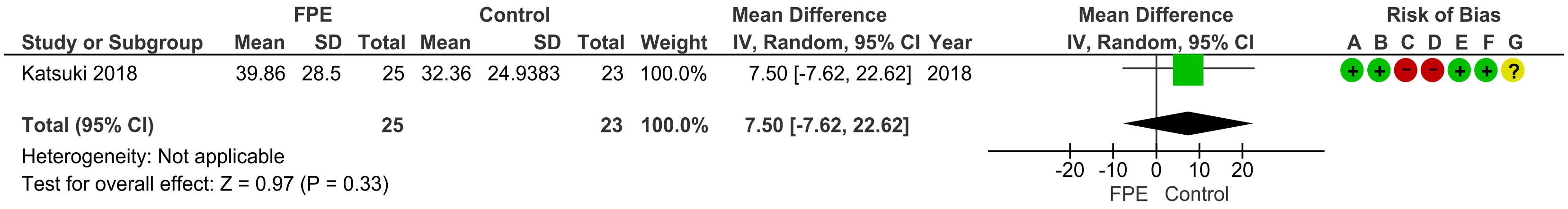

Supplementary Figure 3. Forest plots for family functioning. (a) at 16 weeks. (b) at a final follow-up

(a)

Study or Subgroup	FPE Mean	FPE SD	FPE Total	Control Mean	Control SD	Control Total	Weight	Std. Mean Difference IV, Random, 95% CI	Year	Std. Mean Difference IV, Random, 95% CI	Risk of Bias A	B	C	D	E	F	G
Kumar 2015	84	8.63	38	76.1	6.01	34	51.4%	1.04 [0.55, 1.54]	2015		−	?	?	?	+	?	?
Katsuki 2018	46.25	7.3	25	44.58	7.2995	24	48.6%	0.23 [-0.34, 0.79]	2018		+	+	−	−	+	+	?
**Total (95% CI)**			**63**			**58**	**100.0%**	**0.64 [-0.15, 1.44]**									

Heterogeneity: Tau² = 0.26; Chi² = 4.56, df = 1 (P = 0.03); I² = 78%
Test for overall effect: Z = 1.58 (P = 0.11)

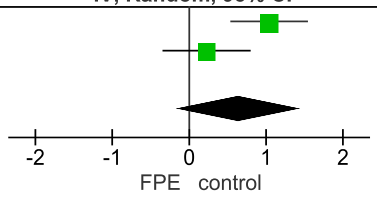

(b)

Study or Subgroup	FPE Mean	FPE SD	FPE Total	Control Mean	Control SD	Control Total	Weight	Std. Mean Difference IV, Random, 95% CI	Year	Std. Mean Difference IV, Random, 95% CI	Risk of Bias A	B	C	D	E	F	G
Seikkula, J 2013	67.3	15.2	29	66.1	17.4	22	32.9%	0.07 [-0.48, 0.63]	2013		?	?	−	−	−	?	?
Kumar 2015	84	8.63	38	76.1	6.01	34	34.5%	1.04 [0.55, 1.54]	2015		−	?	?	?	+	?	?
Katsuki 2018	45.54	10.85	25	45.2	10.8386	23	32.6%	0.03 [-0.54, 0.60]	2018		+	+	−	−	+	+	?
**Total (95% CI)**			**92**			**79**	**100.0%**	**0.39 [-0.28, 1.06]**									

Heterogeneity: Tau² = 0.28; Chi² = 9.35, df = 2 (P = 0.009); I² = 79%
Test for overall effect: Z = 1.15 (P = 0.25)

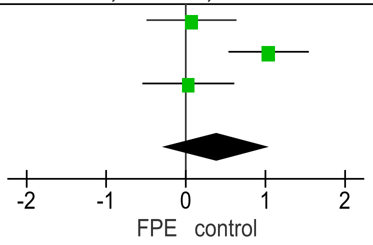

Supplementary Figure 4. Forest plots for patients' general functioning. (a) at 16 weeks. (b) at a final follow-up

(a)

Study or Subgroup	FPE Mean	FPE SD	FPE Total	Control Mean	Control SD	Control Total	Weight	Mean Difference IV, Random, 95% CI	Year
Katsuki 2018	8.49	10	25	10.34	8.6325	23	100.0%	-1.85 [-7.12, 3.42]	2018
**Total (95% CI)**			**25**			**23**	**100.0%**	**-1.85 [-7.12, 3.42]**	

Heterogeneity: Not applicable

Test for overall effect: Z = 0.69 (P = 0.49)

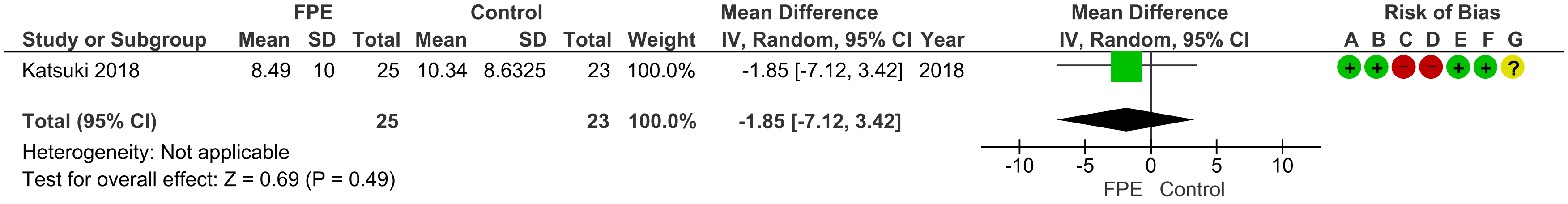

(b)

Study or Subgroup	FPE Mean	FPE SD	FPE Total	Control Mean	Control SD	Control Total	Weight	Mean Difference IV, Random, 95% CI	Year
Katsuki 2018	9.36	10.5	25	8.41	9.1121	23	100.0%	0.95 [-4.60, 6.50]	2018
**Total (95% CI)**			**25**			**23**	**100.0%**	**0.95 [-4.60, 6.50]**	

Heterogeneity: Not applicable

Test for overall effect: Z = 0.34 (P = 0.74)

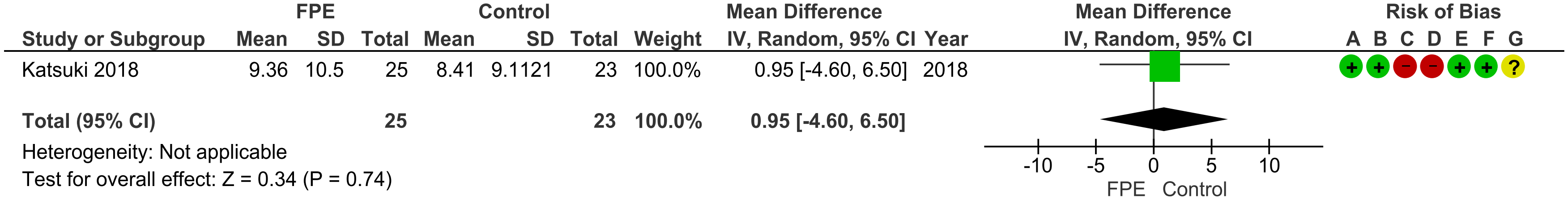

Supplementary Figure 5. Forest plots for family members' distress. (a) at 16 weeks. (b) at a final follow-up

(a)

Study or Subgroup	FPE Events	FPE Total	Control Events	Control Total	Weight	Risk Ratio M-H, Random, 95% CI	Year
Seikkula, J 2013	6	35	9	31	100.0%	0.59 [0.24, 1.47]	2013
**Total (95% CI)**		**35**		**31**	**100.0%**	**0.59 [0.24, 1.47]**	
Total events	6		9				
Heterogeneity: Not applicable							
Test for overall effect: Z = 1.13 (P = 0.26)							

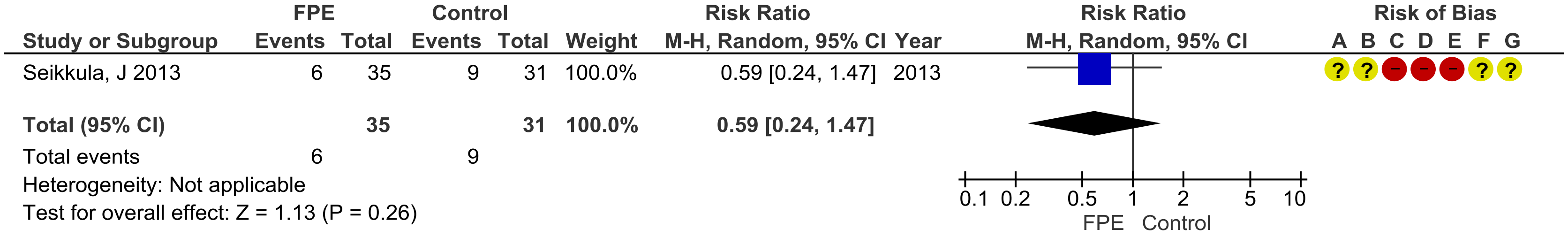

(b)

Study or Subgroup	FPE Events	FPE Total	Control Events	Control Total	Weight	Risk Ratio M-H, Random, 95% CI	Year
Lemmens 2009 (multifamily)	8	35	6	12	32.1%	0.46 [0.20, 1.05]	2009
Lemmens 2009 (Single-family)	6	25	6	12	27.5%	0.48 [0.20, 1.18]	2009
Seikkula, J 2013	6	35	9	31	26.6%	0.59 [0.24, 1.47]	2013
Kumar 2015	2	40	6	40	9.4%	0.33 [0.07, 1.55]	2015
Hinton 2020	2	15	1	8	4.4%	1.07 [0.11, 10.04]	2020
**Total (95% CI)**		**150**		**103**	**100.0%**	**0.50 [0.31, 0.80]**	
Total events	24		28				
Heterogeneity: Tau² = 0.00; Chi² = 0.89, df = 4 (P = 0.93); I² = 0%							
Test for overall effect: Z = 2.89 (P = 0.004)							

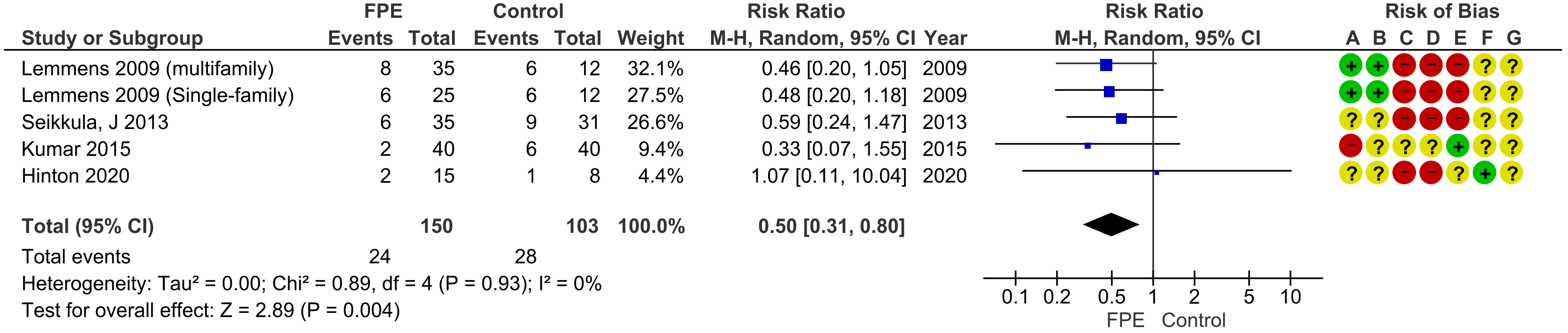

Supplementary Figure 6. Forest plots for dropout rate. (a) from intervention. (b) from assessment.
