# Supplementary figures and images for: Effectiveness of family psychoeducation for major depressive disorder: systematic review and meta-analysis
Source: BJPsych Open. 2022 Aug 2;8(5):e148. doi: 10.1192/bjo.2022.543 (PMC9380172; doi:10.1192/bjo.2022.543)

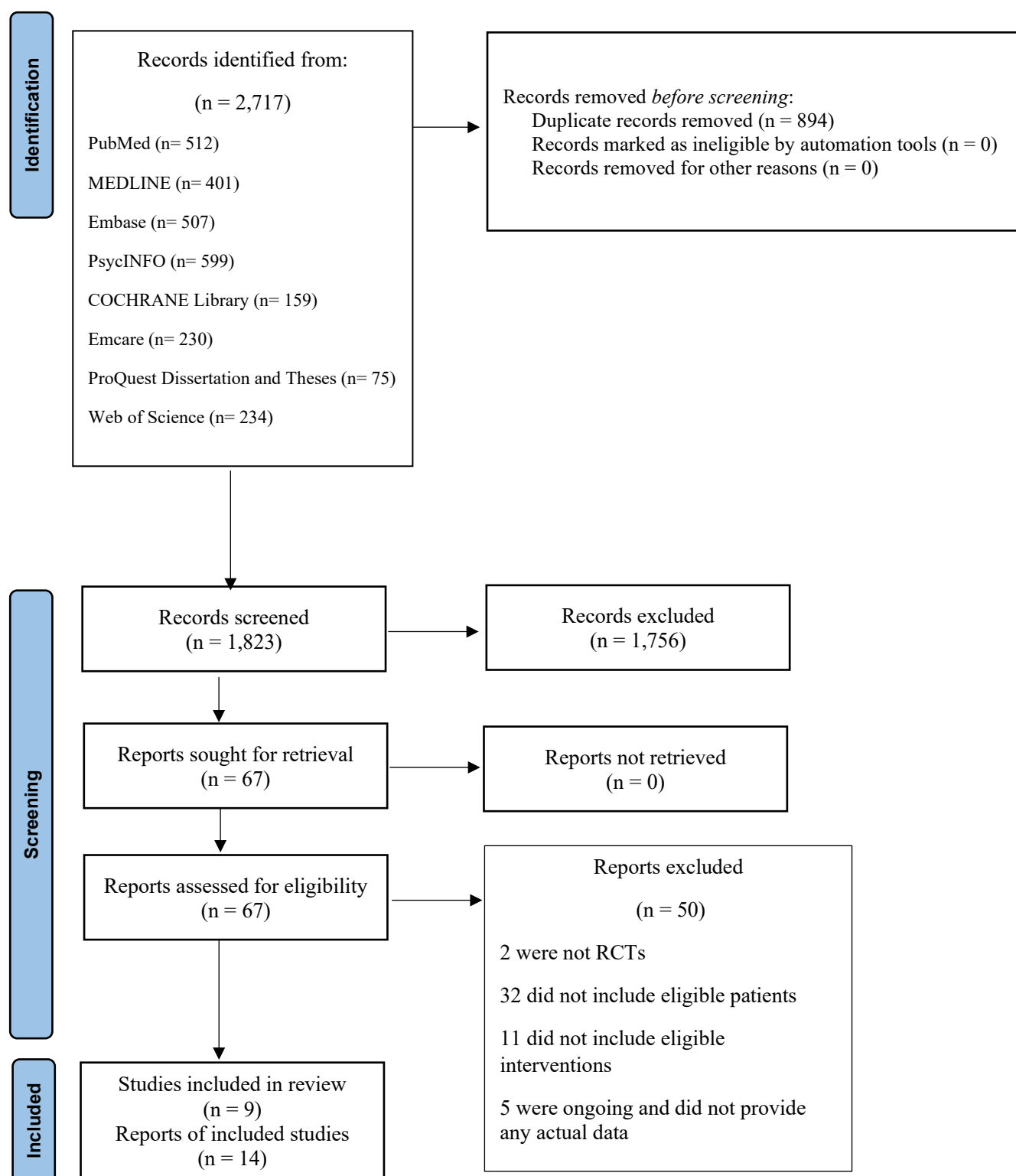

Supplementary Figure 1. Flowchart for inclusion of studies.

Supplement: Supplementary file 1 [file S2056472422005439sup001.zip › Supplementary Figure 1.pdf]
